# Gender differences in elementary school students’ fraction learning: roles of spatial ability and mathematical anxiety

**DOI:** 10.3389/fpsyg.2024.1464501

**Published:** 2024-12-12

**Authors:** Ruhai Zhang, Zhongting Chen, Ciping Deng

**Affiliations:** ^1^Shanghai Key Laboratory of Brain Functional Genomics, Affiliated Mental Health Center (ECNU), School of Psychology and Cognitive Science, East China Normal University, Shanghai, China; ^2^Shanghai Changning Mental Health Center, Shanghai, China

**Keywords:** gender difference, spatial ability, mathematical anxiety, fraction learning, mediation effect

## Abstract

**Aim:**

In this study, we examined gender differences in fraction learning and explored potential underlying mechanisms.

**Methods:**

The mediating effects of spatial ability and mathematical anxiety on gender differences in fraction learning were tested in elementary school students. A total of 165 sixth-grade students (83 girls) from public elementary schools participated in the study. All participants completed a series of tasks, including a mathematical anxiety test, two spatial ability tasks (spatial working memory and mental rotation tests), and a fraction knowledge test (incorporating fraction arithmetic, fraction number line, and fraction concept knowledge).

**Results:**

Significant gender differences exist in student performance in terms of fractional arithmetic, fractional number line estimation, mathematical anxiety, and spatial working memory. We also identified a chain-mediating effect of spatial working memory and mathematical anxiety on the relationship between gender and fractional arithmetic.

**Conclusion:**

The findings suggest a developmental pathway linking gender differences in spatial cognition to fraction learning, contributing to a better understanding of the cognitive and affective factors underlying gender differences in fraction learning during early adolescence. Furthermore, there are several practical implications for reducing gender differences in mathematics education during school years.

## Introduction

The existence of gender differences in mathematics learning has been a long-standing controversy, influenced by specific mathematical domains and the developmental stages at which examinees are examined (Gibbs, 2010; Hutchison et al., 2019; Hyde et al., 2008; Lindberg et al., 2010). In most cases, men and women perform similarly in mathematics, although specific domains may exhibit substantial gender differences that favor men (Else-Quest et al., 2010; Lindberg et al., 2010). For instance, few gender differences have been observed in basic numerical skills and simple arithmetic problem solving (Hutchison et al., 2019); however, men and women show significant differences in complex problem solving (Lindberg et al., 2010).

The extent of these differences may be influenced by the relationship between mathematics domains and spatial ability (Bull et al., 2013; Geary et al., 2021a, 2023). A recent meta-analysis showed that spatial and mathematical abilities are consistently related (Atit et al., 2022). This relationship can be attributed to several factors, such as the mental representation of number information and problem-solving strategies (Delgado and Prieto, 2004; Mix, 2019; Mix and Cheng, 2012). People with better spatial abilities can solve problems using more efficient visuospatial representations of problems or overlapping brain activations between spatial and mathematical abilities (Dumontheil and Klingberg, 2012; Hubbard et al., 2005; Zago et al., 2008). Men generally outperform Women in most spatial domains, and this advantage increases with age, as reflected in both spatial and mathematical performance differences (Hyde et al., 1990; Lauer et al., 2019; Voyer et al., 2017).

**Figure 1:**
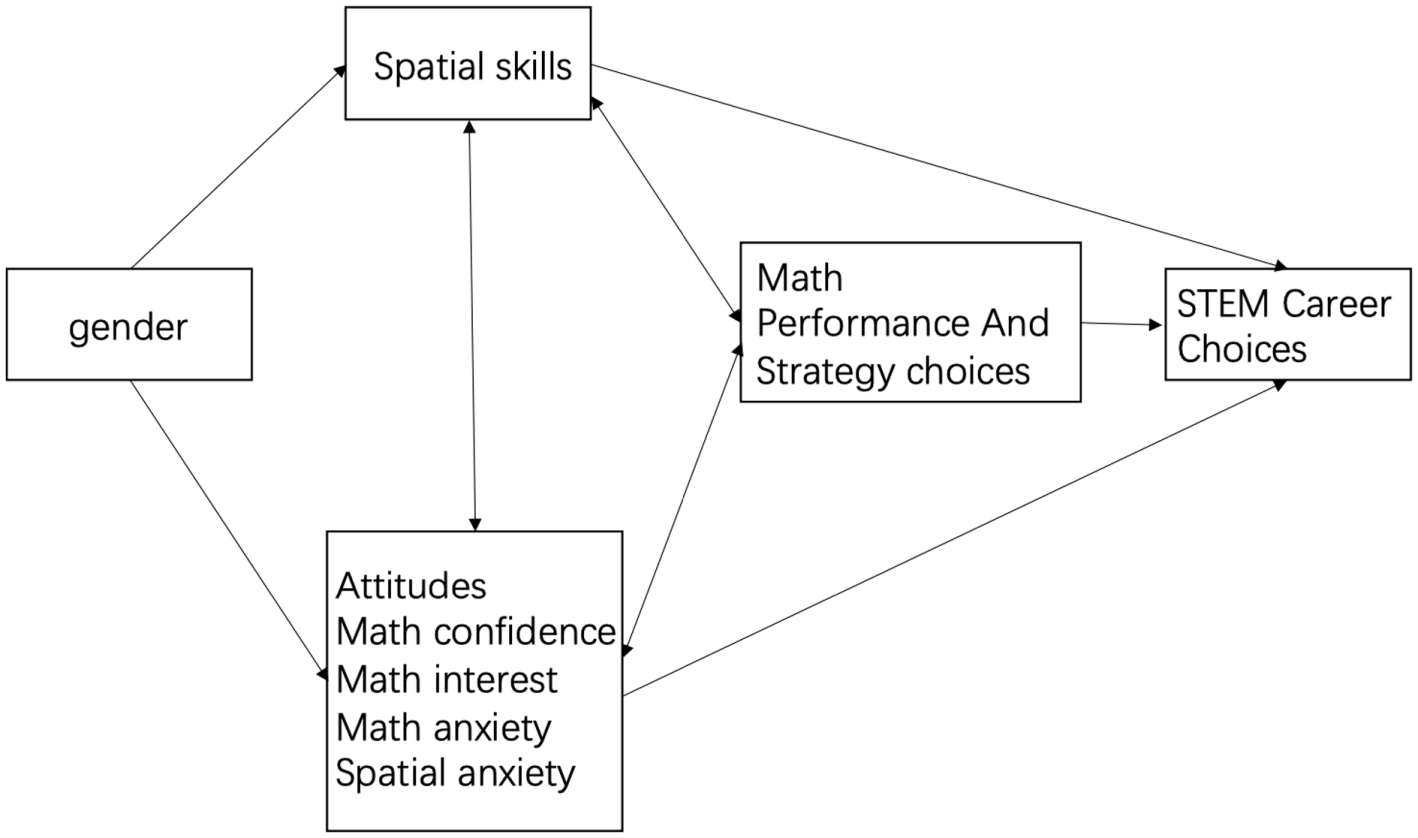
A bio-Psycho-Social model proposed by Casey and Ganley (2021).

Mathematical anxiety may play a mediating role in gender differences in mathematical performance as it is correlated with performance and has a negative relationship with spatial skills (Barroso et al., 2021; Ferguson et al., 2015; Maloney, 2011; Namkung et al., 2019; Zhang et al., 2019). Boys tend to have lower mathematical anxiety (Geary et al., 2021a; Stoet et al., 2016). Casey and Ganley (2021) proposed a biopsychosocial model explaining the interplay between spatial ability, mathematical anxiety, and gender differences in mathematical performance (Figure 1). Wang (2020) also proposed a sequential mediation model that allows the simultaneous testing of two mediational relationships. In this model, the paths begin from gender to spatial ability and mathematical anxiety, both of which affect mathematical achievement. However, currently, there is little evidence to support this model.

This study examined the extent of gender differences in fraction learning among 6th-grade students in Chinese elementary schools. Adolescence is a crucial period for the development of gender differences in spatial-mathematics domains. Research has shown that the extent of gender differences in spatial abilities and certain areas of mathematics tended to increase during this period (Hyde et al., 1990; Lauer et al., 2019). The study of gender differences in fractions may be an important window for verifying how spatial ability and mathematical anxiety explain gender differences in mathematics according to the biopsychosocial model. Fractions were selected as a focus of study owing to their importance in primary school mathematics learning and their association with spatial skills (Geary et al., 2023; Meert et al., 2009; Sidney et al., 2019).

### Fraction learning

Fraction is not only fundamental to children’s mathematical development (Bailey et al., 2012; Siegler et al., 2012) but also plays a pivotal role in shaping their future success in science, technology, engineering, and mathematics related domains (Sadler and Tai, 2007). Elementary school students’ proficiency in fractions serves as a strong predictor of their forthcoming mathematics scores and acquisition of algebraic skills (Booth and Newton, 2012; Booth et al., 2014; Siegler et al., 2012). Additionally, most individuals (more than two-thirds) reported incorporating fractions into their daily work routines (Handel, 2016).

However, many students and adults find it difficult to learn fractions (Sidney et al., 2019). Unlike whole-number procedural knowledge, multiplication in fractions cannot be understood solely as a repeated addition; multiplication and division in fractions can result in either a larger or smaller number. Siegler and Pyke (2013) discovered that sixth-grade students were correct on only 41% and eighth-grade students on 57% of fractional arithmetic problems. Despite years of experience learning mathematics, many adults continue to face significant challenges in understanding fractions. Even educated adults, such as pre-service teachers and middle school students, are prone to misunderstanding concepts related to fractional multiplication and division. For example, many incorrectly believe that multiplying fractions always increases value, and dividing fractions always decreases it, a misconception rooted in whole-number arithmetic thinking (Siegler and Lortie-Forgues, 2015). Siegler and Lortie-Forgues (2015) found that pre-service teachers correctly judged fraction multiplications with values less than one approximately 33% of the time, and the accuracy for fraction division was even lower (30%). This suggests that even when adults demonstrate procedural proficiency in fraction calculations, they may lack a conceptual understanding of fraction operations.

Fractional competencies can be categorized into two main types: conceptual and procedural knowledge (Bailey et al., 2014; Rittle-Johnson et al., 2001). Conceptual knowledge involves understanding the nature and mathematical properties of fractions, such as their relationships (ratio/rate/proportion), operation, and measurement aspects (fraction conceptual knowledge) (Obersteiner et al., 2019). The magnitude of a fraction is affected by the relationship between its numerator and denominator, and the magnitudes can be arranged in a specific order on a number line. This understanding of fraction magnitude is part of the conceptual knowledge associated with fractions (Hecht and Vagi, 2010; Jordan et al., 2013; Siegler et al., 2013; Vamvakoussi and Vosniadou, 2004). Alternatively, procedural knowledge refers to understanding arithmetic algorithms that can be applied to fractions and the ability to use them accurately during problem-solving (Bailey et al., 2015).

### Gender, spatial abilities, and fraction learning

Spatial ability plays a crucial role in learning fractions. Young children grasp fractional concepts through spatial proportional reasoning (Congdon et al., 2018; Möhring et al., 2016). Comprehending units and their representations of equal spatial divisions form the foundation for grasping the concept of fractions. Children who possess a stronger grasp of the relative sizes of proportions can more easily visualize fractions through spatial analogies (Möhring et al., 2016). This enhanced spatial understanding can subsequently aid in understanding numerical fractions. Spatial analogies is significant in students’ understanding of fractional magnitudes (Fuchs et al., 2013). Furthermore, fractions can be represented on a mental number line using spatial formats (Mix, 2019; Mix and Cheng, 2012). Moreover, fractional procedural knowledge tasks require more complex spatial operations than whole-number procedural knowledge problems, such as rotation, separation, and recombination of varying amounts (Mix et al., 1999). For example, when students calculate 13/8 × 2/3, noticing the shared factor “2″ between the denominator “8″ and the numerator “2″ allows them to reduce the problem to 13/4 × 1/3 (cross-reduction strategy), which could be easier to solve. Problems with larger operands tend to be solved less accurately than those with smaller operands (Zbrodoff and Logan, 2005).

Boys tend to outperform girls in fractional tasks (Bailey et al., 2014; Geary et al., 2021b; Hallett et al., 2012; Resnick et al., 2016). Additionally, boys generally demonstrate stronger spatial skills than girls (Casey, 2013; Lauer et al., 2019; Voyer et al., 2017), which is significant because spatial skills are closely linked to fraction learning. Men’s advantage in various spatial domains (Lauer et al., 2019; Voyer et al., 2017), combined with the connection between spatial ability and fractional performance, provides a plausible explanation for why boys and men often excel in fractions (Geary et al., 2021b). However, spatial abilities are not the only factor influencing this gender gap; mathematical anxiety is also correlated with fractional performance, suggesting a more complex interaction (Sidney et al., 2019).

### Gender, mathematical anxiety, spatial ability, and fraction learning

Mathematical anxiety refers to feelings of fear, tension, and worry experienced by young students and adults when engaging in mathematics (Ashcraft, 2002). High mathematical anxiety is associated with lower mathematical achievement (Ma, 1999). This can lead to the avoidance of mathematics classes and hinder opportunities to improve mathematical skills, resulting in a negative relationship between mathematical anxiety and achievement (Hembree, 1990). The transition from learning whole numbers to fractions often intensifies anxiety and fear during primary schooling (Sidney et al., 2019). Individuals with high mathematical anxiety may struggle with learning fractions (Rayner et al., 2009; Starling-Alves et al., 2022).

In previous studies, researchers have also found gender differences in mathematical anxiety (Devine et al., 2012; Hart and Ganley, 2019), and spatial ability may play an important role in these differences (Ferguson et al., 2015; Sokolowski et al., 2019). Although there are minimal gender differences in mathematical performance (Else-Quest et al., 2010), girls tend to report higher levels of mathematical anxiety than boys. However, the exact reason for the discrepancy remains unclear. Gender differences in mathematical anxiety may stem from the social stereotype that women are worse at mathematics than are men (Beilock et al., 2007; Goetz et al., 2013).

Alternatively, gender differences in mathematical anxiety may be mediated by spatial processing abilities (Maloney et al., 2012). Specifically, lower spatial processing abilities in girls and women may have contributed to higher mathematical anxiety. Gender differences in spatial abilities result in increased spatial anxiety, which in turn influences overall anxiety toward mathematics (Ferguson et al., 2015; Sokolowski et al., 2019). If students experience anxiety about spatial reasoning, they may also feel anxious about mathematics in general because many mathematical problems require spatial reasoning or strategies (Sokolowski et al., 2019). Gender differences in spatial abilities emerge earlier than gender differences in mathematical anxiety. Moore and Johnson (2008) observed gender differences in spatial abilities as early as 5 months of age, and these differences persisted after childhood (Halpern et al., 2007; Mortensen et al., 2003). However, gender differences in mathematical anxiety are observed in the late elementary and middle school stages (Devine et al., 2012; Else-Quest et al., 2010; Hart and Ganley, 2019). Therefore, considering the relationship between spatial abilities and mathematical anxiety, as well as the order in which they occur in students, lower spatial abilities may be one of the causes of mathematical anxiety. Moreover, spatial abilities and mathematical anxiety may jointly explain gender differences in fraction learning.

Casey and Ganley (2021) identified four primary factors that significantly influence gender differences in mathematical performance within their biopsychosocial model: (1) gender-based variations in brain organization patterns; (2) gender differences in spatial skills; (3) a strong correlation between spatial and mathematical abilities, supported by both behavioral research and overlapping brain structures; and (4) gender differences in mathematical attitudes and anxieties. These factors are influenced by biological, psychological, and sociocultural factors. Boys’ biological inclination toward spatial interests and their increased engagement in such activities compared to girls contribute to the widening of the initial gender gap in spatial abilities (Sherman, 1978). Casey (1996) underscored the intricate interplay between biological predispositions (brain organization and innate preferences) and sociocultural factors (attitudes, societal pressures, experiences, and discrimination). These factors interact significantly to shape individual differences within and between genders. Based on the previous literature, it is evident that spatial ability and mathematical anxiety may not solely account for gender differences in mathematical performance (Ferguson et al., 2015; Maloney et al., 2012; Sokolowski et al., 2019).

A comprehensive review of research exploring gender differences in spatial ability, mathematical anxiety, and fractions amalgamated various findings to elucidate the connections between these variables. Through this synthesis, Wang (2020) proposed a sequential mediation model that allows for the simultaneous testing of two mediational relationships. This model includes paths from gender to spatial ability, mathematical anxiety, and mathematical achievement through deductive reasoning.

### Current study

In the present study, we investigated gender differences in fraction learning, the role of spatial ability, and mathematical anxiety in sixth graders who had recently completed formal instruction during the winter of sixth grade. Examining fraction competencies becomes particularly critical at the sixth grade level, as it frequently represents the final year during which students receive comprehensive instruction in fractions in Chinese elementary schools (Ministry of Education, 2012).

**Figure 2:**
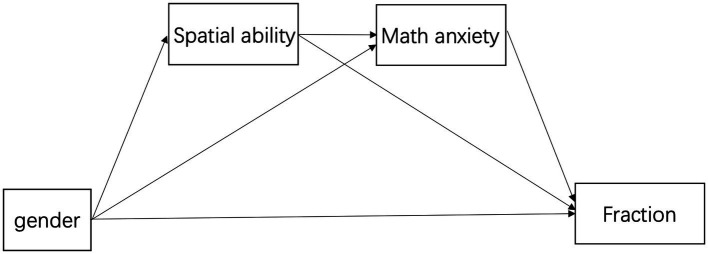
Gender-spatial ability-math anxiety-fractions model.

The biopsychosocial model proposed by Casey and Ganley (2021) and sequential mediation model proposed by Wang (2020) were considered for our model. We proposed a similar model to explain gender differences in fraction learning during early adolescence with a focus on spatial ability and mathematical anxiety (Figure 2).

The main hypotheses of the current study were as follows: (1) boys would outperform girls in fractions and spatial ability tasks and exhibit lower levels of mathematical anxiety; (2) higher mathematical anxiety would have a negative relationship with both fractions and spatial abilities; (3) higher spatial abilities would have a positive relationship with fractions; and (4) spatial ability and mathematical anxiety would mediate the relationship between gender and fraction learning. The sequential mediation model was as follows: gender- > spatial ability- > mathematical anxiety- > fraction learning.

## Method

### Participants

Participants were recruited from two public elementary schools in a major city in Anhui, China, where most children came from families with moderate socioeconomic backgrounds. These schools typically represent families with average socioeconomic conditions in such regions. While we did not collect detailed income data, this demographic is characterized by average parental education levels (generally high school or vocational school) and occupations common in county-level cities (e.g., small business owners, service workers, or agricultural workers).

They were 165 sixth graders (mean age = 11 years and 9 months, SD = 0.44 years, 82 girls), who did not experience any intellectual, sensory, or behavioral difficulties (according to their teachers’ report). Participants with known intellectual, sensory, or behavioral difficulties were excluded to ensure the validity of our findings. Intellectual difficulties were defined as significant limitations in cognitive functioning, such as reasoning and problem-solving, typically diagnosed through standardized assessments. Sensory difficulties refer to impairments in vision, hearing, or other sensory modalities that can affect students’ abilities to perceive and interact with the environment. Behavioral difficulties included persistent issues, such as ADHD or emotional disorders, which might interfere with the ability to focus or follow instructions. Teachers familiar with students’ academic and social behaviors provided reports of any such difficulties, as no formal diagnostic assessments were conducted. This exclusion was important to avoid confounding factors that could affect cognitive tasks central to our study, such as spatial ability and mathematical anxiety.

We used the G*Power software to assess the sample size of the direct effect in a multiple regression model with four predictors, aiming for a power of 80%. Assuming an effect size of f^2^ = 0.11, the analysis indicated a minimum requirement of 114 students.

### Measures

#### Mathematical anxiety test

Mathematical anxiety was assessed using the Mathematics Anxiety Scale for Children (Chiu and Henry, 1990). The assessment lasted approximately 10 min and used a pen and paper. Two items that may lead to confusion in China were excluded (i.e., “listening to another student explain a mathematical problem” and “using the tables in the back of a mathematics book”). Chinese students mainly rely on teacher-centered learning with limited opportunities for collaborative learning, leading to fewer opportunities for students to listen to their peers explaining mathematical problems. This item caused confusion among many students during the preliminary experiment. Additionally, Chinese mathematics textbooks lack tables in the back, thus we removed the item “using the tables in the back of a mathematics book.” Participants completed the test in class, rating their anxiety levels for each statement on a 4-point Likert-type scale ranging from 1 (not nervous) to 4 (very nervous). The instructions were read to the participants, followed by 20 items (e.g., taking a mathematics quiz). When requested, the participants were provided with clarification of any item. Possible scores ranged from 20 to 80 points. Higher scores indicated higher levels of anxiety. The maximum and minimum scores for mathematical anxiety were 66 and 20, respectively. The Cronbach’s alpha was 0.90 for this test.

#### Spatial skills

Considering the distinct relationships between different spatial abilities and mathematics (Casey and Ganley, 2021), we used two spatial ability tests: a mental rotation task and a visuospatial working memory task.

#### Mental rotation task

Mental rotation is a fundamental aspect of spatial ability that involves mentally rotating objects within two-dimensional or three-dimensional space. This capacity enables individuals to visualize the appearance of an object after rotation, making it crucial for completing multistep spatial visualization tasks. We used a 2D mental rotation task as a measure of students’ mental rotation ability. The Mental Rotation Test was conducted using a computerized visual task, where the letter “R” was presented in various orientations (0°, 45°, 90°, 135°, 180°, 225°, 270°, and 315°), either in its normal or mirrored form—adapted from Dalecki et al. (2012). The task was administered on a computer using E-Prime 3.0 software. Each stimulus was presented three times across 48 trials. The participants were asked to decide whether the letter was normal or mirrored. Each trial began with a fixation cross displayed for 500 ms, followed by a 100 ms pause. The test stimulus then appeared on the screen and remained until the participant responded or for a maximum of 5 s. Participants were instructed to decide as quickly as possible whether the stimulus was “normal” or “mirrored.” They pressed the “F” key for “normal” items and the “J” key for “mirrored” items. No feedback was provided during the trials. The final score was calculated as the proportion of correctly identified items. Therefore, a higher score indicated better performance. The accuracy of the test ranged from 19 to 100%. Cronbach’s alpha was.89 for this test.

#### Spatial working memory

Spatial working memory, which refers to the capacity to retain and manipulate visual images over short periods, is an essential component of spatial ability. This type of memory is crucial for multistep spatial visualization and mental rotation mathematical problems. The Corsi Block-Tapping Task (Kessels et al., 2000) was used to assess spatial working memory. The task was administered on a computer using E-Prime 3.0 software. Students were presented with a display of nine squares (3 × 3 cell matrix), and one cartoon-frog jumped among the squares, one by one, in any of the squares. The participants were required to recall the sequence of squares in which the frog jumps in the correct order by clicking on the screen with a mouse. The sequence length started with two squares and increased to nine squares. Students made three attempts for each sequence length. If one of the sequences was recalled correctly two or more times, the next sequence level was initiated; if three sequences at the same level were recalled correctly only once and below, the task was terminated. The highest level of the sequence was used as the final score. Thus, a higher score indicated better performance. The scores for this item ranged 2–9. Cronbach’s alpha was.78 for this test.

### Fraction competency

#### Fraction procedural knowledge task

Participants completed 31 fractional arithmetic problems to test their fraction procedural knowledge. We adapted materials from Hecht (1998). First, we converted the mixed numbers into improper fractions in the addition and subtraction problems, which increased the probability of simplification and added complexity to the calculations. Second, we added two multiplication problems with the same denominators. Multiplication problems with the same denominators may mislead students into using the approach typically applied in fraction addition problems (e.g., 7/8 × 3/8 = 7 × 3/8). Finally, we included three division problems involving fractions, two with the same denominators and one with different denominators, ensuring an equal number of problems with the same and different denominators. The set of items included 6 addition (e.g., 2/5 + 1/ 5), 6 subtraction (e.g., 3/4–1/4), 11 multiplication (e.g., 7/8 × 2/5), and 8 division (e.g., 1/3 ÷ 4) problems. In all fractional arithmetic tasks, 15 items required simplification or could be solved using cross-reduction strategies (for details, please see the supplement). Each item appeared individually on a computer screen. Participants were provided with scratch papers to use while computing answers, but entered the answers via a computer keyboard, with no time limit. Students were instructed that the final answer must be in its simplest form; otherwise, they would be considered incorrect. Fractions equivalent to the correct answers were scored as correct in the simplest form. Thus, higher scores indicated better performance. The accuracy of the test ranged from 20 to 100%. Cronbach’s alpha was 0.94 for this test.

#### Fraction conceptual knowledge task

The participants’ fractional conceptual knowledge was assessed using a version corresponding to their grades. It combined items from previous studies (Hallett et al., 2012), and was assessed using a paper-and-pencil task to measure sixth-grade fraction knowledge. This task consisted 20 items assessing different aspects of fraction conceptual understanding, such as part-whole meaning (e.g., shade 2/5 on a picture portioned into 10 equal pieces), ratio meaning (a point at which two fractions show a higher value), and common fraction concepts such as “how many possible fractions are between 1/4 and 1/2?” A higher score indicated better performance. The maximum score for this item was 20 and the minimum score was 8. Cronbach’s alpha was 0.65 for this test.

#### Fraction 0–5 number line estimation

We used the fraction 0–5 Number Line Estimation to measure students’ knowledge of fraction magnitude representation. Participants viewed 10 lines on a computer screen one at a time, as adapted from Geary et al. (2021b). Each line’s left endpoint was labeled “0,” and the right endpoint was labeled “5.” The fraction that participants estimated in each trial was presented above the middle of the number line.

Each fraction was from a different tenth of the number line and the numerators and denominators were selected to limit the correlations between the magnitudes of the fractions and their components. The fractions were 1/19, 4/7, 7/5, 13/9, 8/3, 11/4, 10/3, 7/2, 17/4, and 9/2. Participants responded by moving the cursor to the position of their desired estimate and clicking on a mouse. Performance was measured as each participant’s mean percentage absolute error (PAE); that is, |R − C| × 0.2, where R = response and C = correct placement. Therefore, a higher score indicated worse performance. The PAE values of the participants ranged from 0.002 to 0.430. Cronbach’s alpha was 0.79 for this test.

### Procedure

This study was approved by the Human Subjects Ethics Subcommittee of East China Normal University and conducted in accordance with the ethical principles of research involving human participants. Students were recruited from their mathematics classrooms with permission from the school; participation was voluntary, and parental consent and student assent were obtained. The tests were administered by two professionally trained master’s and doctoral psychology students with extensive experience in conducting such assessments. After informed consent for participation was obtained, the tests were administered to groups (approximately 15 students at a time) in a multimedia classroom during winter in sixth grade. All tests lasted for approximately an hour. The test results were not shared with the students or teachers. We organized a psychology seminar focused on mental health as a token of appreciation for the participating students.

### Data analysis

All statistical analyses were conducted using SPSS 22 and Mplus version 8.0. Descriptive statistics and bivariate correlations were calculated to summarize the sample’s performance across key measures, including fractional arithmetic, fractional number line estimation, fractional conceptual knowledge, spatial working memory, mental rotation, and mathematical anxiety. Considering the differential relationship between the components of spatial ability and mathematics (Casey and Ganley, 2021), we studied mental rotation and visuospatial working memory separately rather than treating them as a whole. In the descriptive analyses, we also examined gender differences in these variables. Additionally, we conducted gender difference tests on the proportion of procedural knowledge items requiring cross-reduction or simplification. This was performed to examine whether items requiring more spatial operations and complex procedural knowledge exhibited gender differences.

Mplus v8 was used to estimate the models by employing a full-information maximum likelihood estimation to handle missing data. We incorporated different types of spatial abilities (mental rotation and visuospatial working memory) and their respective relationships with mathematical anxiety and fraction knowledge into mediation models to explore potential gender differences. We examined the hypothesis concerning the sequential mediating effect of spatial ability—specifically, mental rotation or visuospatial working memory—and mathematical anxiety on the relationship between gender and fraction learning. Indirect effects were assessed using bias-corrected bootstrapped 95% confidence intervals (95% CIs) based on 5,000 samples.

## Results

### Descriptive analysis and gender differences

Descriptive statistics by gender are presented in Table 1. Boys significantly outperformed girls on the spatial working memory task and the 0–5 number line task, whereas girls reported higher levels of mathematical anxiety. Boys performed better than girls on fractional arithmetic, although the effect was marginal (*p* = 0.051, *d* = 0.32). Additionally, boys outperformed girls on fractional arithmetic items that required reduction or simplification (e.g., 13/8 × 2/3 = 26/24 marks as incorrect; *t* (163) = 2.69, *p* < 0.01, *d* = 0.42). However, there were no gender differences when the number of correct answers did not need to be simplified in fractional arithmetic [e.g., 13/8 × 2/3 = 26/24 mark as correct; *t*(163) = 1.20, *p* = 0.23, *d* = 0.15]. However, no gender differences were observed in the mental rotation and fractional conceptual knowledge tasks.

**Table 1:** Descriptive statistics: means, standard deviations, and effect sizes for gender differences.

	Boy		Girl				
	*M*	*SD*	*M*	*SD*	*t*	*p*	Cohen’s *d*
FK	17.72 (*N* = 81)	2.31	17.65 (*N* = 75)	2.14	0.21	0.83	0.03
MA	31.35 (*N* = 83)	8.80	34.71 (*N* = 82)	10.17	−2.27	0.03	−0.35
NL05	11.49 (*N* = 82)	10.23	15.83 (*N* = 74)	12.07	−2.41	0.02	−0.39
FA	0.82 (*N* = 83)	0.15	0.77 (*N* = 82)	0.16	1.97	0.05	0.32
SWM	5.69 (*N* = 83)	1.10	5.32 (*N* = 82)	1.06	2.19	0.03	0.34
MR	0.77 (*N* = 72)	0.18	0.79 (*N* = 72)	0.14	−0.83	0.41	−0.12

FK, fraction concept knowledge; MA, math anxiety; NL05, fraction number line 0–5; FA, fraction arithmetic; SWM, spatial working memory; MR, mental rotation.

### Correlations analysis

Table 2 presents the correlations between the variables. Fractional arithmetic was related to fractional knowledge, mental rotation, and the fractional number line task. Fractional concept knowledge was significantly related to spatial working memory, mental rotation, and the fractional number line. The fractional number line was significantly related to spatial working memory but not to mental rotation or mathematical anxiety. These correlations revealed that gender, spatial working memory, mathematical anxiety, and fractional arithmetic were all significantly correlated.

**Table 2:** Bivariate correlations among variables.

	Gender	FK	MA	NL05	FA	SWM
FK	−0.017					
MA	**0.175** ^*^	−0.138				
NL05	**0.192** ^*^	**−0.166** ^*^	0.065			
FA	**−0.152** ^**†**^	**0.442** ^**^	**−0.196** ^**^	**−0.162** ^*^		
SWM	**−0.169** ^*^	**0.241** ^**^	**−0.199** ^**^	**−0.162** ^*^	**0.195** ^**^	
MR	0.069	**0.212** ^**^	−0.025	−0.071	**0.285** ^**^	**0.153** ^*^

FK, fraction concept knowledge; MA, math anxiety; NL05, fraction number line 0–5; FA, fraction arithmetic; SWM, spatial working memory; MR, mental rotation; *p < 0.05; **p < 0.01; ^†^*p* < 0.1.

### Mediation analysis

Considering the gender differences in spatial working memory and its relationship with mathematical anxiety and fractional knowledge, we included spatial working memory separately in the model. Specifically, we examined the significance of the serial indirect path in the model of gender, spatial working memory, mathematical anxiety, and fractional arithmetic using current data.

**Figure 3:**
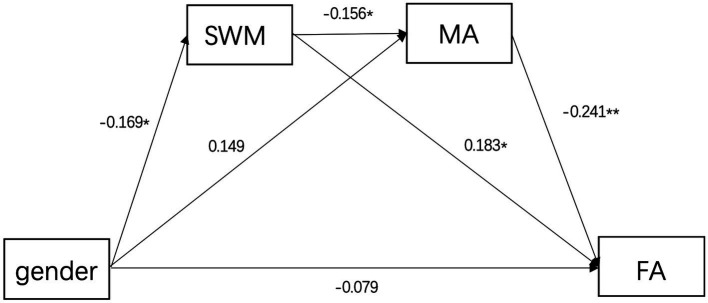
Gender Differences in Fraction Arithmetic Mediated by SWM and MA. MA, math anxiety; FA, fraction arithmetic; SWM, spatial working memory. *p < 0.05;** *p* < 0.01.

The results revealed that the total indirect effect was significant [effect = −0.073, 95% CI (−0.149, −0.022)], accounting for 48% of the total effect. The specific indirect effects are shown in Figure 3. The indirect effect of spatial working memory was significant [effect = −0.031, 95% CI (−0.095, −0.001)], suggesting that spatial working memory mediates the relationship between gender and arithmetic fraction. Similarly, the indirect effect of mathematical anxiety was significant [effect = −0.036, 95% CI (−0.091, −0.002)], suggesting that mathematical anxiety mediates the relationship between gender and fraction arithmetic. The combined indirect effect of spatial working memory and mathematical anxiety was also significant [effect = −0.006, 95% CI (−0.020, −0.001)]. These indirect effects accounted for 23.7 and 20.4% (spatial working memory and mathematical anxiety individually), and 3.9% (both sequentially) of the total effect, respectively. Spatial working memory and mathematical anxiety fully mediated the gender differences in fractional arithmetic.

Furthermore, we examined whether spatial working memory mediated gender differences in fractional number line estimation. The findings revealed that the indirect impact of spatial working memory was not significant [95% CI (−0.001, 0.159)].

## Discussion

In this research, we examined gender differences in fraction learning among sixth graders and explored the influence of spatial ability and mathematical anxiety. Sixth grade, the time for pupils to learn a fraction, is a key period for examining competence in fractions, given its significance in mathematics learning and relevance to future science, technology, engineering, and mathematics education (Bailey et al., 2012; Siegler et al., 2012). Therefore, understanding gender differences in fraction learning is crucial. This investigation was motivated by previous research that suggested a link between low spatial ability, mathematical anxiety (Ashkenazi and Danan, 2017; Ferguson et al., 2015; Maloney et al., 2012), and poor mathematical achievement (Ashcraft and Kirk, 2001; Ashcraft and Ridley, 2005; Imbo and Vandierendonck, 2007; Maloney, 2011). In this study, we also provide experimental evidence for the models proposed by Wang (2020) and Casey and Ganley (2021), offering insights for a deeper understanding of these models.

To develop educational programs that address the gender gap in fraction learning, it is critical to comprehend the potential mediating chain from gender to fractions involving spatial ability and mathematical anxiety. The findings of this study highlight the significant roles played by spatial ability and mathematical anxiety in fraction learning. The results provide evidence for the mediating relationship between gender and spatial working memory, mathematical anxiety, and fractional arithmetic (marginal significance).

### Spatial ability, mathematical anxiety, and fraction learning

Researchers have identified various connections between spatial abilities and diverse aspects of mathematical knowledge (Hegarty et al., 2006; Linn and Petersen, 1985; Mix and Cheng, 2012; Uttal et al., 2013). In fractions, fractional arithmetic requires more complex spatial operations such as rotation, separation, recombination of varying amounts. Additionally, spatial working memory and mental rotation are crucial in this process (Mix et al., 1999). Spatial working memory may aid students in improving their number line estimation performance by allowing them to consider locations associated with specific numbers (Gunderson and Hildebrand, 2021).

Students often experience heightened mathematical anxiety when learning fractions, a more advanced and conceptually challenging area of mathematics. We found that mathematical anxiety was significantly associated with both fractional arithmetic and conceptual knowledge, but not with fractional number line tasks in the sixth grade. This finding aligns with previous research that has addressed the relationship between mathematical anxiety and fraction learning (Rayner et al., 2009; Sidney et al., 2019; Starling-Alves et al., 2022). This difference can be attributed to the distinct cognitive demands of each task. According to Starling-Alves et al. (2022), mathematical anxiety primarily disrupts symbolic fraction tasks, which are more cognitively demanding, because they rely on working memory resources. Conversely, nonsymbolic ratio tasks, such as those involving line length comparisons, are less affected by mathematical anxiety owing to their more intuitive processing requirements (Starling-Alves et al., 2022). Sidney et al. (2019) further highlighted that mathematical anxiety reduces accuracy in fraction comparison and estimation tasks, as these require specific cognitively taxing calculations. However, fractional number line tasks demand a more holistic spatial estimation of magnitude than symbolic calculation, thereby minimizing the impact of anxiety on performance (Sidney et al., 2019). Mathematical anxiety impairs both procedural and conceptual knowledge of fractions, as both involve complex reasoning and thus consume significant cognitive resources, making them more susceptible to the effects of anxiety (Rayner et al., 2009). In summary, mathematical anxiety predominantly affects tasks that are symbolically complex and conceptually demanding, whereas tasks requiring intuitive processing, such as number line tasks, remain relatively unaffected.

Importantly, mathematical anxiety can manifest as early as elementary school, as noted by Szczygieł and Pieronkiewicz (2022), who found that it is negatively correlated with mathematical performance in young children. They identified time pressure, task difficulty, and fear of failure as primary contributors to mathematical anxiety, which in turn diminished children’s ability to succeed in mathematical tasks. This early emergence of mathematical anxiety has a lasting impact as it consumes cognitive resources—particularly, working memory—which are crucial for handling complex mathematical tasks (Ashcraft, 2002). According to the Processing Efficiency Theory (Eysenck and Calvo, 1992) and Attentional Control Theory (Eysenck et al., 2007), anxiety reduces the efficiency of working memory, thereby impairing students’ ability to manage the cognitive demands of complex mathematical tasks. Students with higher levels of mathematical anxiety struggled with both the procedural and conceptual aspects of fractions.

In addition, Moustafa et al. (2020) proposed a neural network model that explains how mathematical anxiety is linked to changes in brain function, particularly through increased activity in the amygdala, which is responsible for processing fear, and reduced inhibition in the prefrontal cortex. This neural disruption intensifies emotional responses to mathematics-related tasks, further hindering students’ cognitive abilities when they engage in mathematics. Such changes in neural activity heighten mathematical anxiety and impair performance, particularly in tasks that require more intricate cognitive operations, such as fractional arithmetic. This understanding is reinforced by Vargas (2021), who reviewed how social and emotional factors, such as the experience of failure, task difficulty, and time pressure, contribute to the intensification of mathematical anxiety. These external pressures exacerbate students’ negative emotions toward mathematics, leading to a further decline in performance.

### Gender differences in fraction learning

Not all aspects of fractional knowledge exhibit gender differences. Boys outperformed girls on fractional arithmetic and fractional number line tasks, whereas no significant gender differences were observed in fractional conceptual knowledge. These results are consistent with those of previous studies (Bull et al., 2013; Geary et al., 2021b; Hallett et al., 2012). These findings are consistent with the view that boys and girls perform similarly on basic mathematical tasks, but boys may perform better in domains that place higher demands on spatial and procedural knowledge (Gibbs, 2010; Lindberg et al., 2010).

### Gender differences in fraction learning: the role of spatial ability

Spatial ability explains gender differences in fractional arithmetic and fractional number line estimations. Distinct relationships between different types of mathematical knowledge and spatial abilities exist (Hegarty et al., 2006; Linn and Petersen, 1985; Mix and Cheng, 2012; Uttal et al., 2013). We found that gender differences exist in spatial working memory, but not in mental rotation. In-depth spatial working memory mediated gender differences in fractional arithmetic performance, but not in fractional conceptual knowledge or fractional number line estimation. These findings align with those of Geary et al., 2021b, who indicated that spatial working memory mediates gender differences in fractional arithmetic. Gender differences in fractional number line estimation can be attributed to disparities in visuospatial attention (Geary et al., 2021b) rather than spatial working memory or mental rotation skills.

Gender differences in fractional arithmetic may stem from the problem-solving strategies employed. We observed notable gender differences in fractional arithmetic tasks, especially in simplification or cross-reduction problems. However, no significant differences were found for tasks in which simplification was unnecessary. We suggest that boys employ more cross-reduction strategies to solve fractional arithmetic problems by leveraging the computational advantages of reduction procedures. Even though students were taught to use cross-reduction strategies to solve these problems, those with low spatial working memory may not have noticed this information between the denominators and numerators, hindering their use of cross-reduction strategies. Further supporting this, previous research has highlighted that spatial ability is crucial for boys’ mathematical development, particularly in areas requiring spatial reasoning. For example, Geary et al. (2023) found that the spatial pathway to mathematical competence is more significant for boys in grades 6 to 9. Similarly, Lombardi et al. (2019) demonstrated that boys who excel in mental rotation tasks show notable improvements in mathematical reasoning, especially in the fifth and seventh grades, whereas girls exhibit no such association. Supporting this, Vasilyeva et al. (2009) found that boys outperformed girls in elementary school measurement tasks requiring spatial reasoning and were more likely to employ spatial strategies such as diagrammatic representations and mental comparisons. By contrast, girls tended to excel in formula-based tasks, relying more on numerical reasoning and algorithms but encountered greater challenges with spatial reasoning, especially when tasks required visualizing or mentally manipulating objects that were not explicitly depicted. Further research is needed to explore the relationship between spatial ability and the use of fractional arithmetic strategies such as cross-reduction.

### Gender differences in fraction learning: the role of mathematical anxiety

Mathematical anxiety is another key factor in gender differences in fractional arithmetic. Several studies have consistently shown that the negative correlation between mathematical anxiety and performance is stronger in girls than boys, contributing to the observed gender differences in mathematical outcomes. For example, Devine et al. (2012) found that, after controlling for test anxiety, only girls displayed a significant negative correlation between mathematical anxiety and performance. This suggests that although boys experience mathematical anxiety, its impact on their performance is less pronounced. Van Mier et al. (2019) further highlighted that, in second- and fourth-grade girls, there was a clear negative correlation between mathematical anxiety and performance, which was not observed in boys. According to Yu et al. (2024), girls are more likely to immerse themselves in emotions such as anxiety when facing mathematical tasks, whereas boys are more likely to use cognitive restructuring strategies to manage their emotions. This emotional sensitivity likely exacerbates the impact of anxiety on girls’ mathematical performance by interfering with their cognitive processing. This indicates that mathematical anxiety may play a prominent role in girls’ mathematical development. In our study, we found that mathematical anxiety significantly mediated the relationship between gender and fractional arithmetic performance. The observed gender differences in mathematical anxiety may be a key factor in explaining why girls underperform in certain mathematical tasks, especially those requiring spatial reasoning, such as fractional arithmetic. Our findings are consistent with prior research indicating that girls tend to internalize negative emotions, including anxiety, more than boys.

However, gender differences do not appear in all types of fractional knowledge nor do they emerge in all studies (Hansen et al., 2015; Ye et al., 2016). The reasons for this could be as follows. First, in types of fraction knowledge that are influenced by classroom attention, girls may compensate for their lower spatial ability with their better classroom attention. For example, girls may have better in-class attention behavior (Geary et al., 2021a), which promotes fraction conceptual knowledge (Vukovic et al., 2014; Ye et al., 2016). Unlike their U.S. counterparts, Chinese teachers emphasize developing both procedural and conceptual understanding by depending on conventional and inflexible techniques, which have demonstrated their effectiveness in imparting mathematical knowledge in the classroom (An, 2008).

Second, the difficulty of fraction-knowledge tests plays a key role. When the test difficulty or complexity is low, gender differences may not appear (Casey and Ganley, 2021; Hyde et al., 2008). More complex mathematical problems reveal gender differences in strategy choices. Researchers have found that women tend to use more conventional strategies even when non-algorithmic methods are easier (Gallagher and De Lisi, 1994). Girls are also more likely to make procedural errors, such as “count all,” when solving “missing number” problems (Hornburg et al., 2017). Strategy choice matters, because some methods are more effective or efficient than others. Similarly, gender differences were more pronounced when testing items that required simplification or cross-deduction strategies. However, when no simplification was required, the gender differences disappeared.

Finally, third, the timing of the fractional knowledge tests is important. Gender differences in fraction knowledge may be more likely to appear during the early stages, when students are just beginning to learn fractions, and these differences may disappear later on (Hallett et al., 2012; Resnick et al., 2016). Many researchers have suggested that girls tend to be at a disadvantage when dealing with novel or newly introduced mathematical content. For example, a detailed analysis of the spatial-mathematics relationship across kindergarten, third, and sixth grades suggested that the association is stronger when students are faced with novel problems and weakens as their skills become more automatic or procedural (Mix et al., 2016; Mix et al., 2017).

### Gender, spatial ability, mathematical anxiety, and fraction learning

The key novel finding of the present study is the mediating pathway from gender to fractional arithmetic performance created by spatial working memory and mathematical anxiety. The results of this study are consistent with and contribute to the ongoing discussion on gender differences in mathematics, particularly within the biopsychosocial frameworks proposed by Casey and Ganley (2021) and Wang (2020). According to these models, cognition and emotion interact to influence mathematical outcomes. Consistent with the biopsychosocial model, our findings reaffirm the significance of spatial working memory and mathematical anxiety in mediating gender differences in fraction learning.

In this study, boys demonstrated stronger spatial working memory than girls were relatively weaker. This sex-based difference was particularly evident in tasks involving fractional arithmetic. Fraction arithmetic problems, such as cross-reduction or simplification, require the use of spatial skills to manage the relationships between fractions (e.g., interactions between numerators and denominators). Weaker spatial working memory limits students’ ability to effectively use these strategies, increasing the complexity of problem solving. Owing to girls’ disadvantages in spatial working memory, this cognitive gap causes them to experience more difficulty when solving fractional problems that require spatial reasoning. As the difficulty increases, students’ anxiety toward the task also increases. Anxiety is often a response to perceived cognitive inadequacy, particularly when performing mathematical tasks that require complex spatial reasoning. Gender differences in mathematical anxiety may be mediated by spatial processing abilities (Ferguson et al., 2015; Maloney et al., 2012; Sokolowski et al., 2019). Specifically, lower spatial processing abilities in girls and women may have contributed to higher mathematical anxiety. When students struggle to solve these problems effectively, a sense of helplessness emerges, which exacerbates their anxiety. This anxiety further limits their ability to process information and impairs their problem-solving efficiency. Increased mathematical anxiety directly affects fractional arithmetic performance. Anxiety consumes working memory resources that are crucial for multistep operations (such as multiplying, dividing, or simplifying fractions). Mathematical anxiety slows students’ problem-solving speed, increases the likelihood of errors, and further impairs their performance on fractional arithmetic tasks. Therefore, deficits in spatial working memory led to higher mathematical anxiety, which, in turn, reduced performance in fractional arithmetic, forming a chain mediation effect.

By combining the pathways of cognitive and affective predictors, we demonstrated that these factors collectively explain a significant portion of the association between gender and fractional arithmetic. The boys’ advantage in spatial ability, coupled with lower mathematical anxiety, provides them with more effective strategies for solving fractional arithmetic problems, particularly those that require spatial reasoning, such as cross-reduction strategies or simplifications. By contrast, girls’ disadvantages in spatial working memory may affect their use of cross-reduction strategies or simplification when solving fractional arithmetic. The utilization of such strategies can effectively reduce the complexity of problems, consequently lowering mathematical anxiety when tackling similar problems and ultimately influencing the resolution of fractional arithmetic problems. This provides critical evidence of a potential link that can be further addressed in experimental studies to establish a causal relationship.

This understanding has practical and educational implications for reducing gender differences in mathematics learning. By focusing on interventions that enhance spatial skills and provide strategies to manage mathematical anxiety, particularly for girls, educators can help bridge the gender gap in procedural fractional knowledge and other mathematics-related areas. Early intervention and targeted support are key to fostering equitable mathematics learning outcomes.

### Cross-cultural variations in gender differences in fraction knowledge

Research on cross-cultural differences in mathematics has highlighted trends in achievement, including gender differences, and the effects of factors such as mathematical anxiety and spatial ability on performance (Else-Quest et al., 2010). Studies indicate that Chinese students generally perform well in international mathematics assessments, often excelling in procedural and conceptual fraction knowledge, compared to their Western counterparts (Bailey et al., 2015; Torbeyns et al., 2015). Notably, gender differences in fractional knowledge between Chinese and American students may be shaped by distinct underlying mechanisms. In the Chinese sample, students tended to use routine algorithms and symbolic representations, whereas the U.S. sample preferred concrete visual representations (Cai, 2000a, 2000b). Various factors influence these problem-solving preferences. For instance, routine algorithm use may be driven by higher levels of mathematical anxiety, because this approach demands more cognitive resources, leading to a higher cognitive load. In contrast, the use of concrete visual representations is likely supported by spatial ability, suggesting that strong spatial skills may influence U.S. students’ preference for visual strategies, potentially enhancing their creativity in problem-solving. These differences in problem-solving approaches may contribute to variations in how Chinese and American students interpret gender differences in fractional knowledge.

This divergence is also reflected in the instructional approaches of Chinese and American teachers, who place distinct demands on students’ abilities. Chinese teachers predominantly use procedural algorithms and symbolic representations, emphasizing rules and calculation skills, thereby prioritizing logical reasoning over spatial understanding and placing less demand on spatial abilities. However, this method can increase cognitive load and mathematical anxiety as it requires intensive cognitive resources(An et al., 2004; Zhou et al., 2006). Conversely, American teachers favor concrete visual representations, such as fraction puzzles, diagrams, and physical models, to facilitate students’ intuitive understanding. This approach relies heavily on spatial abilities, which support an intuitive grasp of fractions and encourage creative problem-solving (An et al., 2004; Zhou et al., 2006). The results showed that spatial ability plays a central role in U.S. fraction instruction, whereas in China, where procedural and symbolic methods are prioritized, spatial ability plays a minor role in learning fractions. These instructional and problem-solving differences may contribute to the interpretation of gender differences in fractional knowledge across cultural contexts.

## Limitations

It is important to note that mediation analysis is correlational; thus, causal relationships cannot be definitively established. Most likely, some of the relationships reported here are bidirectional, such as the relationship between mathematical anxiety and fraction learning. Conceptually, mediation offers a potential mechanism underlying the relationship between predictors and outcomes (Dearing and Hamilton, 2006). Establishing this relationship is critical for investigating gender differences in fractions. However, to rigorously test the relationships revealed in this study, future research could use experimental or longitudinal designs to examine whether spatial working memory and mathematical anxiety causally influence differences in fraction learning outcomes.

We assessed the students’ mental rotation ability using 2-D materials. It is possible that the mental rotation task we used was too easy for sixth-grade students, resulting in no observed gender differences. Future studies could use 3-D mental rotation tasks or tasks with greater difficulty to explore whether task complexity influences the observed relationships. Considering that Geary et al. (2021b) found no significant relationship between 3-D mental rotation and fraction learning, future research should also investigate the relative contributions of 2-D and 3-D spatial tasks in predicting fraction learning outcomes. These extensions would clarify the role of spatial ability in fraction learning and its interaction with mathematical anxiety.

Notably, we did not control for IQ or verbal working memory, both of which may correlate with spatial ability and serve as consistent predictors of mathematics achievement (Bull and Lee, 2014; Deary et al., 2007; Geary et al., 2017; Swanson and Sáez, 2003). Future studies could address this limitation by including measures of IQ and verbal working memory to disentangle their effects on fraction learning and spatial ability.

Additionally, owing to the challenges of conducting this study during the COVID-19 pandemic, data collection was particularly difficult, resulting in a sample size of only 165 participants. The limited sample size reduced the statistical power to detect indirect effects. Future research with larger and more diverse samples would provide a more robust test of the hypothesized relationships.

Despite these limitations, we successfully explored the relationships between spatial ability, mathematical anxiety, and fractions, along with the associated gender differences, which had not been previously investigated. These findings contribute to a nuanced understanding of gender differences in fractional arithmetic, suggesting that such differences may arise due to gender disparities in spatial working memory and mathematical anxiety. Future research could further explore these mechanisms to develop targeted interventions aimed at reducing gender gaps in mathematical performance.

## Data Availability

The raw data supporting the conclusions of this article will be made available by the authors, without undue reservation.
