# Supplementary material for: Gender differences in elementary school students’ fraction learning: roles of spatial ability and mathematical anxiety
Source: Front Psychol. 2024 Dec 12;15:1464501. doi: 10.3389/fpsyg.2024.1464501 (PMC11669514; doi:10.3389/fpsyg.2024.1464501)
Supplement: Supplementary file 1 [file Table_1.docx]

Fraction procedural knowledge tasks

| Fraction arithmetic items | Items needed to simplify or could be used by cross-deduction |
| --- | --- |
| 2/5 + 1/5= |  |
| 3/4 - 1/4= | √ |
| 3/6 + 1/6= | √ |
| 5/6 - 2/6= | √ |
| 13/4 - 1/4= | √ |
| 3/4 + 2/4= |  |
| 33/8 + 12/8= |  |
| 22/3 - 11/3= |  |
| 5/6 + 2/3= | √ |
| 7/8 - 1/2= |  |
| 11/3 - 4/5= |  |
| 3/4 + 2/3= |  |
| 3 × 1/3= |  |
| 40 × 1/2= |  |
| 4 × 4/5= |  |
| 6 × 3/4= | √ |
| 7/8 × 3/8= |  |
| 3/7 × 5/7= |  |
| 7/8 × 2/5= | √ |
| 5/6 × 3/4= | √ |
| 22/3 × 1/2= | √ |
| 13/8 × 2/3= | √ |
| 21/3 × 33/8= | √ |
| 1/3 ÷ 4= |  |
| 1/6 ÷ 3= |  |
| 2 ÷ 3/4= |  |
| 7 ÷ 1/2= |  |
| 3/4 ÷ 1/8= | √ |
| 2/5 ÷ 4/9= | √ |
| 3/8 ÷ 5/8= | √ |
| 10/9 ÷ 5/9= | √ |
|  |  |
